# Presence of interstereocilial links in waltzer mutants suggests *Cdh23* is not essential for tip Link formation

**DOI:** 10.1016/j.neuroscience.2008.10.012

**Published:** 2009-01-23

**Authors:** A.K. Rzadzinska, K.P. Steel

**Affiliations:** Wellcome Trust Sanger Institute, Wellcome Trust Genome Campus, Hinxton, Cambridge, CB10 1SA, United Kingdom

**Keywords:** hair cell, stereocilia, cadherin23, signal transduction, hearing loss, Cdh23, cadherin23, OTOTO, osmium tetroxide/thiocarbohydrazide, PCR, polymerase chain reaction, SEM, scanning electron microscopy

## Abstract

Cadherin23 has been proposed to form the upper part of the tip link, an interstereocilial link believed to control opening of transducer channels of sensory hair cells. However, we detect tip link-like links in mouse mutants with null alleles of *Cdh23*, suggesting the presence of other components that permit formation of a link between the tip of one stereocilium and the side of the adjacent taller stereocilium.

Inner ear sensory hair cells are equipped with an array of mechanosensory stereocilia arranged in rows of graded height. The overall coherence of the bundles of stereocilia is maintained by side-to-side interstereocilial links termed horizontal top connectors in adult animals, but during development cohesion is facilitated by a range of transient lateral links, including shaft connectors and ankle links (Goodyear et al., 2005; Nayak et al., 2007). In addition to these links, tip links joining the tip of a shorter stereocilium with the side of the adjacent taller stereocilium are widely believed to control opening of the transducer channel, increasing the probability of channel opening when the tip link is tensed by deflection of the stereocilia bundle during sound and balance reception (Pickles et al., 1984). We know the identity of only a handful of the molecules involved in the formation, maintenance and function of stereocilia bundles (Nayak et al., 2007). Several studies have suggested that cadherin23 may be directly involved in formation of interstereocilial links (Siemens et al., 2004; Michel et al., 2005). Moreover, a recent report concluded that the tip link is formed from cadherin23 extending from the taller stereocilium, linking with protocadherin15 reaching from the tip of the shorter stereocilium (Kazmierczak et al., 2007). This would imply an identical length of all tip links. However, two independent studies showed that tip link length varies from 90 nm to 190 nm (Furness et al., 2008; Auer et al., 2008). We found tip link-like links and horizontal connectors in mouse mutants devoid of cadherin23, suggesting that this molecule may not be essential to form a structural interstereocilial link.

## Experimental procedures

We examined hair cells from mice with two different mutant alleles of *Cdh23* together with wild type and heterozygote controls. The waltzer mutation (*Cdh23*^*v*^) introduces a stop codon and is predicted to be a functional null allele (Di Palma et al., 2001a) while the waltzer 2J mutation (*Cdh23*^*v2J*^) leads to aberrant splicing and no protein can be detected with an antibody to the intracellular domain of cadherin23 (Michel et al., 2005; Di Palma et al., 2001b). We used the osmium tetroxide/thiocarbohydrazide (OTOTO) processing method (Hunter-Duvar, 1978) to avoid coating artifacts and field emission scanning electron microscopy (Hitachi FESEM 4800 operated at 5 kV, Hitachi High-Technologies, Maidenhead, Berkshire, United Kingdom). Osmium tetroxide was obtained from Agar Scientifics (Stansted, Essex, United Kingdom), thiocarbohydrazide was obtained from Sigma-Aldrich (Pool, Dorset, United Kingdom). Waltzer and waltzer 2J homozygous mice and their heterozygous littermate controls were genotyped using PCR (Holme and Steel, 2002) and sequencing respectively. We analyzed *Cdh23*^*v/v*^ (*n*=3), *Cdh23*^*+/v*^ (*n*=2), *Cdh23*^*v2J/v2J*^ (*n*=7) and *Cdh23*^*+/v2J*^ (*n*=3) mice at P4; and *Cdh23*^*v/v*^ (*n*=1), *Cdh23*^*+/v*^ (*n*=1) and wild type controls (*n*=5) on mixed genetic background at P28. All animal procedures complied with UK Home Office guidelines, UK Animals (Scientific Procedures) Act of 1986. The minimum numbers of animals sufficient to allow appropriate statistical analyses were used, and all possible steps were taken to minimize animal suffering.

## Results

**Fig. 1:**
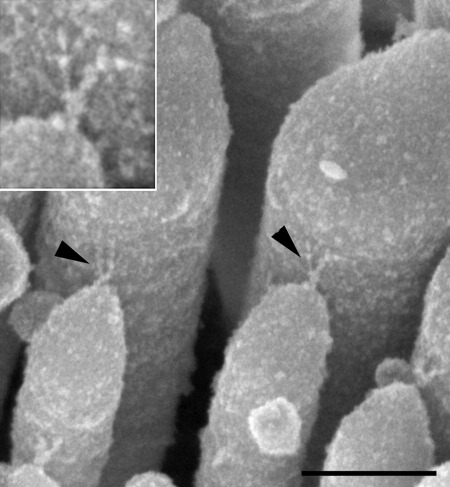
Scanning electron micrographs showing the tip links in control mice. High-resolution image showing stereocilia tips and tip links between inner hair cells stereocilia of control mice. Arrowheads indicate auxiliary links connecting the upper portion of a tip link to the taller stereocilia, the auxiliary link appears thinner than tip link itself. Inset shows higher magnification of one of tip links. Scale bar=250 nm.

Although high-resolution images of tip links in other species have been analyzed and published before (Hackney and Furness, 1995; Kachar et al., 2000; Furness et al., 2008), there is little information on very high-resolution analysis of the normal adult mouse tip link (Furness et al., 1989). In adult guinea pigs, a percentage of tip links have been detected with a forked appearance, consisting of a single lower component branching into two upper components (Hackney and Furness, 1995; Kachar et al., 2000). The forked tip link attachment was shown using OTOTO-S.E.M. (Hackney and Furness, 1995) and freeze fracture imaging techniques but the fragility of this link prevented analysis of the diameter of the attachment arms (Kachar et al., 2000). However, Kachar and colleagues (2000) reported that one of the branches is often retracted. Recently Auer et al. (2008) have used electron tomography to show that the bullfrog tip link also consists of a main link and a thinner upper attachment link, called by the authors an auxiliary link. We found some tip links on control mouse outer hair cells at P28 showed a forked appearance, with two processes extending to the taller stereocilium from a single basal extension from the tip of the shorter stereocilium (Fig. 1). Several of obtained images showed that the two upper processes did not have an identical appearance (Fig. 1) but these links are too small and too fragile to measure their diameter. The data of others and our own results indicate that an additional component may be present in the tip link, rather than the simple model of the tip link being formed of two components alone (cadherin23 and protocadherin15) (Kazmierczak et al., 2007; Corey, 2007). Alternatively, the upper tip link attachment arms could be composed of one (thinner, auxiliary link) and two (thicker arm) filaments, and this explanation also would be in agreement with the electron tomography data on frog hair cells (Auer et al., 2008).

**Fig. 2:**
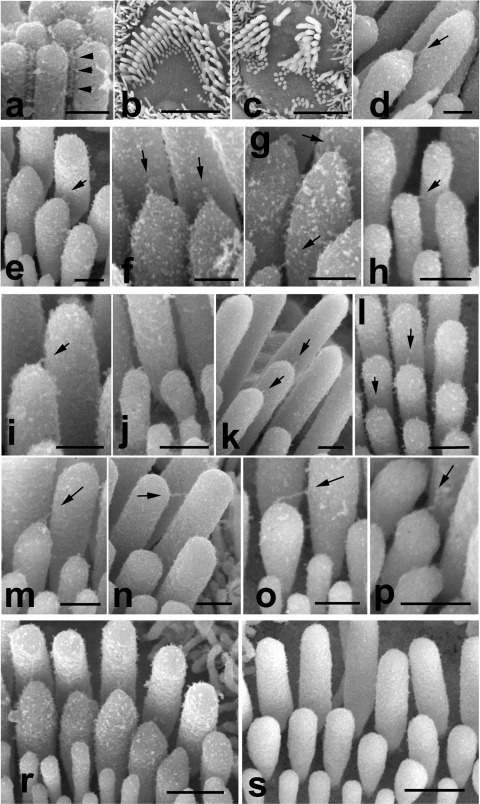
Scanning electron micrographs showing the morphology of stereocilia bundles and interstereocilial links in *Cdh23*^*v*^ and *Cdh23*^*v2J*^ mice. (a) P28 *Cdh23*^*v/v*^ outer hair cell. Arrowheads indicate lateral links within a cluster of mutant stereocilia. (b) P4 *Cdh23*^*+/v*^ outer hair cell, showing normal V-shaped array of stereocilia. (c) P4 *Cdh23*^*v/v*^ outer hair cell, showing disorganized stereocilia bundle. (d) P4 *Cdh23*^*v/v*^ inner hair cell, with tip link indicated by arrow. (e–p) High-resolution images showing representative selection of tip links (arrows) found between stereocilia of *Cdh23*^*+/v2J*^ (e–h) and *Cdh23*^*v2J/v2J*^ (i–p) mice at P4. Note that in j, two links originating at the tips of the shorter stereocilia are attached to the side of the same taller stereocilium. (r, s) Stereocilia bundles on inner hair cells of *Cdh23*^*+/v2J*^ (r) and *Cdh23*^*v2J/v2J*^ (s) at P4, showing reduced tenting of stereocilia tips in the mutant. Scale bars=2 μm (b, c); 250 nm (a, d, e–p); 500 nm (r, s).

In adult *Cdh23*^*v/v*^ mice, horizontal top-connectors are present despite the severe disorganization of the bundle (Fig. 2a) but no tip links could be detected and almost all stereocilia showed rounded tips (data not shown). In order to establish whether the lack of tip links at P28 is caused by cadherin23 deficiency or is a result of progressive degenerative changes affecting stereocilia bundles, we analyzed younger samples, at P4. Stereocilia bundles of *Cdh23*^*v/v*^ and *Cdh23*^*v2J/v2J*^ mice were already disorganized and often split into several clusters by this stage (Holme and Steel, 2002). However, within each coherent cluster the mutant stereocilia, like those of littermate control mice, were graded in height and linked to each other by side-to-side links (data not shown) and tip link-like filaments (Fig. 2b–p). Immature hair bundles show extensive interstereocilial links that could obscure the identification of the oblique links that appear to represent tip links and appear shortly before the onset of FM1-43 dye loading of hair cells and detection of transduction currents (Nayak et al., 2007). Therefore, we focused our analysis upon regions along the length of the cochlear duct where most of the transient lateral links have already gone, leaving a clearer image of the links connecting the tips of shorter stereocilia with the sides of longer stereocilia (Waguespack et al., 2007). We observed a variety of tip-link-like structures in both control and mutant hair bundles from inner and outer hair cells, ranging from very short to very long (Fig. 2d–p) which is consistent with recent data of Furness et al. (2008)). Multiple tip-link-like links were observed in almost every stereocilia bundle in both mutants. However, none of approximately 400 imaged tip-link-like links within control or mutant hair bundles had a forked appearance, which may be related to the developmental properties of a tip link or can be simply be due to a tissue preparation artifact. Shorter stereocilia often have pointed tips, thought to result from membrane tenting caused by the pulling force of the tip link, and in control basal turn inner hair cells, 90% of stereocilia in the second tallest row showed these pointed tips (Fig. 2r) (16.62±1.84 pointed tips out of 18.41±1.68 per inner hair cell, *n*=29). In contrast, in *Cdh23*^*v2J/v2J*^ mutant inner hair cells, only 23% of second row stereocilia showed pointed tips (Fig. 2s) (3.31±2.89 pointed tips out of 14.38±1.70 per inner hair cell, *n*=32).

## Discussion

We clearly show the presence of oblique links between the tips of shorter stereocilia and the sides of longer stereocilia in cadherin23-deficient hair cells during bundle development. The appearance of these links closely resembles the previously publish S.E.M. images of tip links (Furness et al., 1989; Hackney and Furness, 1995; Kachar et al., 2000). We cannot rule out the possibility that the mutants analyzed here have traces of functional cadherin23 capable of forming tip links, although both appear to be null alleles and we observe multiple tip-link-like links within each mutant bundle. However, even if cadherin23 is a component of the tip link as proposed (Siemens et al., 2004; Kazmierczak et al., 2007; Corey, 2007), our observations suggest that it may not be required to form a complete structural link that has an appearance similar in many respects to that of a tip link. Therefore our observations suggest that there may be further, as yet unidentified, molecular components of tip links.
